# Learning from mothers' success in breastfeeding maintenance: coping strategies and cues to action

**DOI:** 10.3389/fpsyg.2023.1167272

**Published:** 2023-05-16

**Authors:** Qiuyan Liao, Jiehu Yuan, Kris Yuet Wan Lok, Siew Fei Ngu, Yuyi Chen, Wendy Wing Tak Lam

**Affiliations:** ^1^School of Public Health, Li Ka Shing Faculty of Medicine, The University of Hong Kong, Pokfulam, Hong Kong SAR, China; ^2^School of Nursing, Li Ka Shing Faculty of Medicine, The University of Hong Kong, Pokfulam, Hong Kong SAR, China; ^3^Department of Obstetrics and Gynaecology, Li Ka Shing Faculty of Medicine, The University of Hong Kong, Pokfulam, Hong Kong SAR, China; ^4^Li Ka Shing Faculty of Medicine, Jockey Club Institute of Cancer Care, The University of Hong Kong, Hong Kong, Hong Kong SAR, China

**Keywords:** cues, positive emotions, qualitative, social support, breastfeeding continuation

## Abstract

This study aimed to gain insight from mothers who were successful in breastfeeding maintenance to develop interventions for promoting breastfeeding maintenance. Following the phenomenological framework, this qualitative study recruited mothers who had maintained breastfeeding for at least 4 months for in-depth interviews. A total of 30 in-depth interviews were completed. We found that almost all participants had experienced an initial adjustment period. During this period, a social support network, personal perseverance in “trying” breastfeeding and “pumping,” and adjusting expectations for breastfeeding to relieve themselves from the pressure of exclusive breastfeeding were important coping strategies. All participants then entered a stage of getting more attuned when breastfeeding was easier. During this period, seeking support from the online mother groups, deliberating medication that might affect breastfeeding, adjusting to accommodate breastfeeding and lives, and managing breastfeeding in public were the main strategies. For working mothers, despite workplace and employers' support, proactive adjustment for using the facilities and lactation breaks for breast milk expression was essential for breastfeeding continuation after returning to work. Throughout the whole journey, positive cues identified from their breastfeeding experiences that helped breastfeeding maintenance included enjoying breastfeeding, breastfeeding as a personal achievement, a healthy and thriving child, positive social feedback, bodily response, the convenience of breastfeeding, and breastfeeding as a motherhood commitment. To conclude, while mothers should be mentally prepared for the difficulties of breastfeeding, they should also be encouraged that things will always get easier as they persevere. Adjustments should be made to accommodate lives and other personal needs. Future studies should consider integrating relevant cues into existing psychosocial interventions for promoting breastfeeding maintenance.

## 1. Introduction

Despite well-established physical, psychological, and social benefits of breastfeeding (Victora et al., 2016; Babic et al., 2020; Hernández-Luengo et al., 2022), exclusive breastfeeding for up to 6 months of an infant's age remained worldwide low (Neves et al., 2021). Even in more developed countries, such as the UK and the US, where initial breastfeeding rates were as high as 80%, the breastfeeding rate declined very fast, being 34% in the UK and 56% in the US for any breastfeeding rates and 1% in the UK and 25% in the US for rates of exclusive breastfeeding, at 6 months postpartum (UNICEF United Kingdom, 2022; US Centers for Disease Control Prevention, 2022).

There have been extensive studies examining the individual and contextual factors that contributed to breastfeeding initiation, continuation, and exclusivity (Balogun et al., 2015; Cohen et al., 2018; Lau et al., 2018; Santana et al., 2018; Patil et al., 2020). Common identified individual factors included maternal socio-demographic factors (e.g., education and employment), smoking status, health conditions, knowledge, perception of breast milk supply, and perceived breastfeeding self-efficacy (Balogun et al., 2015; Cohen et al., 2018; Lau et al., 2018; Santana et al., 2018; Patil et al., 2020). A variety of contextual factors were also identified including social support, medical and healthcare-related factors, and sociocultural factors (Balogun et al., 2015; Cohen et al., 2018; Patil et al., 2020). Existing interventions for promoting breastfeeding outcomes primarily focused on addressing the identified individual and contextual barriers, comprising professional support and education, training on breastfeeding skills, strengthening family, peer, and workplace support, facilitating goal setting, and encouraging to promote self-efficacy (Meedya et al., 2017; Davie et al., 2020; Cordell and Elverson, 2021). However, it was suggested that while education and social support interventions were effective in promoting breastfeeding initiation, they had no or limited effects on promoting breastfeeding continuation (Meedya et al., 2017; Davie et al., 2020; Cordell and Elverson, 2021).

Several knowledge gaps in existing studies may hinder the further development of effective strategies for promoting breastfeeding continuation. First, existing interventions mainly targeted the main psychological constructs for motivating breastfeeding intention, such as knowledge, attitudes, subjective norms, and perceived self-efficacy, or addressed the difficulties in breastfeeding initiation (Meedya et al., 2017; Davie et al., 2020; Cordell and Elverson, 2021). There remains limited understanding about what helps to sustain breastfeeding once breastfeeding is initiated. In addition, existing studies had an emphasis on the barriers to breastfeeding and the social support needed (Stewart-Knox et al., 2003; McInnes and Chambers, 2008; Desmond and Meaney, 2016). Such discourse portrays breastfeeding mothers as vulnerable individuals who merely await help or support and hence underestimate their autonomy in exploring and developing their strategic coping plans throughout the breastfeeding journey (Zhang et al., 2018; Jacobzon et al., 2022). Furthermore, breastfeeding was mainly portrayed as a challenging and effortful behavior rather than a joint reciprocal activity that enables mutual positive affective exchange for both the infant and the mothers (Flacking et al., 2021; Jacobzon et al., 2022). The emotions embodied in mothers' breastfeeding experience (e.g., enjoyment and pride) that could automatically help maintain breastfeeding were generally understudied (Russell et al., 2022). Recent studies started to focus on exploring mothers' positive experiences with breastfeeding and the positive emotions that would help to maintain breastfeeding (Lyons et al., 2019; Flacking et al., 2021; Jacobzon et al., 2022). However, these studies were either related to the experiences of special groups (preterm infants or obese mothers) (Lyons et al., 2019; Flacking et al., 2021) or inadequate in-depth exploration (Jacobzon et al., 2022).

Hong Kong is a metropolis with a highly dense population, where the area of housing per capita is about 16.0 square meters (Hong Kong Census Statistics Department, 2023). Due to the limited land used for properties, infrastructure, and facilities, facilities for breastfeeding in public places and the workplace are highly insufficient (Tarrant et al., 2002). In addition, women in Hong Kong have become increasingly important in the workforce. It was estimated that more than 60% of married women at the age of childbirth were employed (Government of Hong Kong, 2022). All these contexts are barriers to breastfeeding. Several policies have been implemented for promoting breastfeeding continuation, including the implementation of the International Code of Marketing of Breast Substitutes, increasing baby-care facilities in public places, promoting breastfeeding-friendly premises and workplaces, extending maternity leaves from statutory 10 weeks to 14 weeks, and implementing Baby-Friendly Hospital Initiates in all main public hospitals with maternity wards (Family Health Service, 2021; The Government of the Hong Kong Special Administrative Region, 2022). Notwithstanding, the 2021 Breastfeeding Survey estimated that although any breastfeeding rate was 77% at 1 month of child's age, it declined to 43% at 6 months of child's age (22% exclusive breastfeeding) in Hong Kong. This qualitative study conducted among mothers who had maintained any breastfeeding for at least 4 months aimed to gain insights into interventions for breastfeeding maintenance particularly for areas with similar contexts as Hong Kong. Our research questions were as follows:

1. What strategies did mothers use to cope with the experienced challenges during their breastfeeding journey?
2. What were the cues that helped to maintain breastfeeding?

## 2. Materials and methods

### 2.1. Study design

This was a qualitative study conducted following a phenomenological framework (Creswell, 2007) because we aimed to reveal the embodied experiences of mothers who had been “successful” in breastfeeding. Due to the relatively low breastfeeding maintenance rates particularly for exclusive breastfeeding in Hong Kong, we defined “success” in breastfeeding more leniently as feeding infants with breast milk exclusively or in combination with formula, at the breast or via bottles, for at least 4 months. This lenient definition was used to allow some flexibility of the person-centered conceptualization of “successful” breastfeeding rather than merely defining the term from the medical perspective (Whipps et al., 2021). Phenomenology is particularly interested in the lived experiences of a person or a group of people (Creswell, 2007), which makes it suitable for understanding how mothers experienced challenges and developed their coping strategies to tackle the challenges, as well as what helped to maintain breastfeeding throughout the journey.

### 2.2. Participants and sampling

Informants were Hong Kong mothers who were aged 18 years or above, had maintained any breastfeeding for at least 4 months, and were able to communicate in Cantonese. To reduce recall bias, participants should not have stopped breastfeeding for more than 12 months before the interview. Other exclusion criteria included having cognitive difficulties in completing the interview or experiencing serious medical or obstetrical complications in their most recent childbirth that may prevent them from breastfeeding. No other exclusion criteria were specifically set for the informants' infants. Eligible informants were identified and recruited through advertisements distributed by the Hong Kong Breastfeeding Mothers' Association (a local organization that provided various prenatal and postnatal supports for Hong Kong mothers' breastfeeding), campus bulk emails, and Facebook. Purposive sampling was used to recruit informants whose backgrounds were heterogeneous in age, educational attainment, income, employment status, number of children, and breastfeeding duration.

### 2.3. Data collection

Semi-structured in-depth interviews were conducted for data collection. The first 18 interviews were conducted face-to-face between September 2019 and January 2020. Thereafter, due to the COVID-19 pandemic, the interviews were changed to telephone-based to address participants' concerns about the pandemic. The interviews were stopped when concepts identified in at least three consecutive interviews were mainly repetitions of those from the previous interviews, suggesting data saturation. A total of 30 interviews were finally completed. The interviewer first introduced herself to the participant and had casual chats with the participant as a warm-up. After obtaining participants' informed consent, each interview started with asking some basic information about the participants' recent childbirth such as delivery mode, gender and age of the child, and duration of breastfeeding. Participants were then invited to describe how their breastfeeding was initiated, the challenges they encountered, and how they overcame those challenges. Subsequently, participants were asked about whether they encountered any new challenges as breastfeeding continued and how they coped with those new challenges. Working mothers were additionally asked about their concerns before returning to work, and challenges experienced after returning to work and the related coping strategies. All participants were asked whether there were moments when they decided to continue or stop breastfeeding and why they eventually decided to continue. During the interviews, open questions were asked as much as possible. Follow-up questions were asked based on participants' statements for further exploration. For interviews that were conducted during the COVID-19 pandemic, participants were additionally asked about the impact of the pandemic on their breastfeeding experiences. An interview guide was provided in Supplementary material. The interview guide was pilot tested before it was formally used for data collection. All interviews were audio-taped. The interviewers took field notes during and after each interview. Each interview lasted for 30–40 min. All interviews were one-to-one.

### 2.4. Data analysis

All interviews were transcribed verbatim and analyzed in NVivo 12.0 using conventional content analysis to directly generate codes from participants' narrative statements (Hsieh and Shannon, 2005). The coding began with reading and re-reading the transcripts to break down the data into meaningful elements or concepts—the initial codes, based on the researcher's interpretation of the data. New codes were allowed to freely emerge from the data. Then, the initial codes were constantly compared to develop more abstract categories and themes that were relevant to the research topics. The first author (a woman, middle-aged, PhD) who had received training in qualitative research was primarily responsible for choosing informants, conducting the interviews, and analyzing the data, but another two co-authors were involved in peer review to check the transcripts and the codes to reach consensus on category and thematic development. In addition, member checking (Birt et al., 2016) was employed by inviting participants to give feedback on the themes, descriptions of the themes, and illustrating quotes to improve the trustworthiness of the findings.

## 3. Results

**Figure 1:**
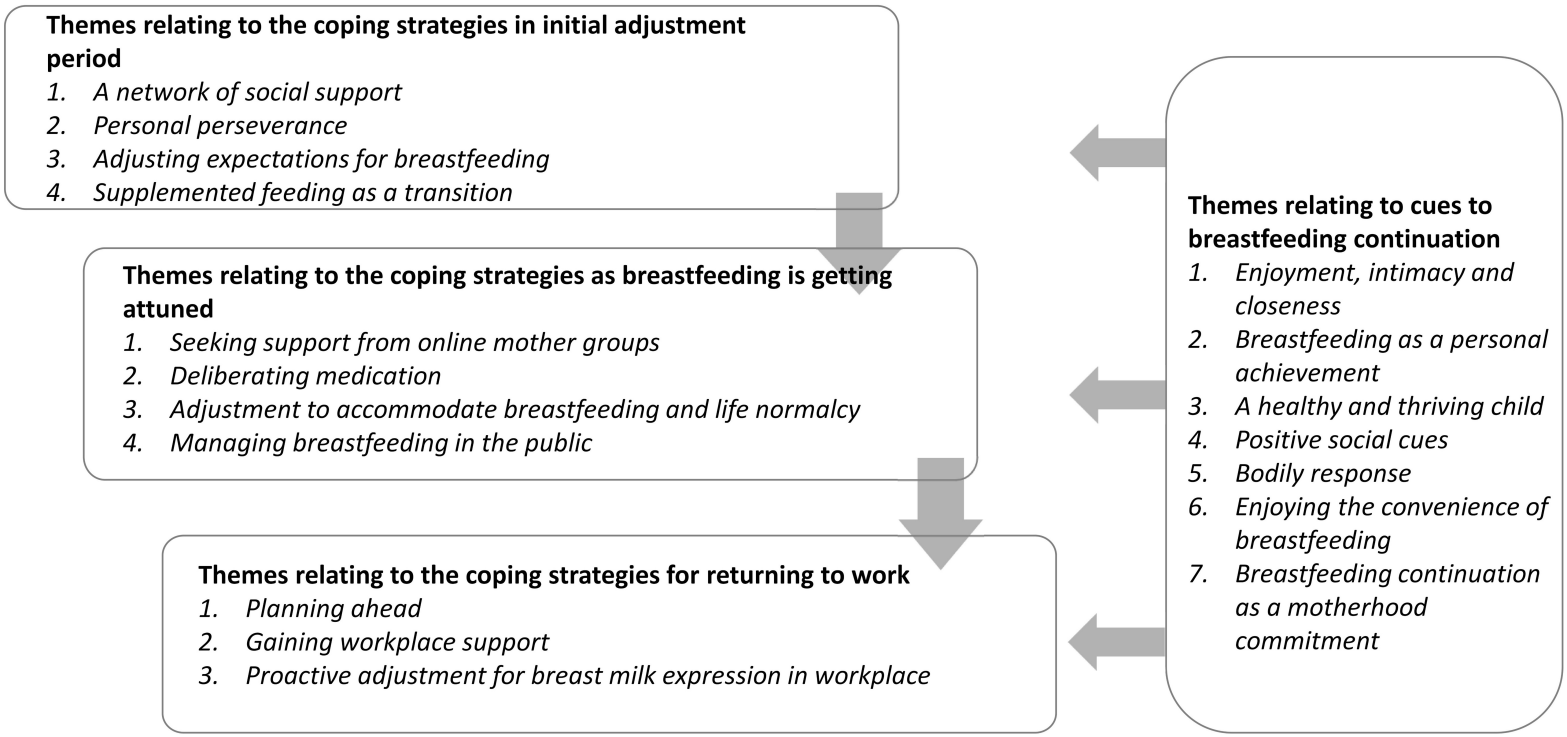
Research themes and their inter-relationships.

The characteristics of the 30 participants are shown in Supplementary material. Of the 30 participants, 6, 17, and 7, respectively, had secondary, tertiary, and master or above educational attainment; nine were unemployed, three had a part-time job, and the remaining had a full-time job. Lengths of breastfeeding ranged from 4 to 22 months. Most (*N* = 27) remained to breastfeed, and the remaining three participants stopped breastfeeding within 6 months of the interview date. More details about participants' characteristics and their child's feeding patterns are shown in Supplementary material. The qualitative data analysis revealed that coping strategies varied and constantly developed by stage of breastfeeding while cues to breastfeeding maintenance were mainly related to participants' positive experiences with breastfeeding which automatically help to maintain breastfeeding throughout their breastfeeding journey. An overview of the themes relating to coping strategies by stage of breastfeeding and cues to breastfeeding continuation is shown in Figure 1. Coping strategies evolved and varied throughout three phases of breastfeeding: initial adjustment, when breastfeeding is getting attuned, and returning to work (for working mothers), while cues to breastfeeding continuation worked to maintain breastfeeding throughout all three phases.

### 3.1. Themes relating to the coping strategies in the initial adjustment period

Almost all participants experienced an initially difficult period in their breastfeeding journey. This period usually lasted for 1–3 months. First-time mothers took a longer time to overcome this period compared with mothers who had prior breastfeeding experience. The main difficulties experienced during this period included child health conditions (e.g., jaundice), infant's refusal to suckle, mothers' inadequate breastfeeding skills, perceived insufficient milk supply, breast problems, physical pain after delivery, unstable moods, and psychological frustration. This is an adjustment period for participants. During this process, participants learned to breastfeed but generally felt uncertain about their capacity in breastfeeding and the feeding cues. Some participants went back and forth to decide whether to breastfeed or use infant formula. For these mothers, using infant formula was always a struggle and a last resort. Four themes emerged relating to coping with the initial challenges: an initial network of social support, personal perseverance, adjusting expectations for breastfeeding, and supplemented feeding as a transition to overcome the initial difficulties.

#### 3.1.1. Initial network of social support

All participants expressed appreciation for various social supports for coping with the initial challenges. These social supports formed a strong network to satisfy mothers' diverse needs regarding breastfeeding initiation, facilitating the learning process, and enhancing their confidence in their capacity for breastfeeding. Specifically, hospital and health professional support helped mothers become knowledgeable, obtain breastfeeding skills, build up confidence in breastfeeding, and address concerns about insufficient feeding.

“*I gave birth in a public hospital, and so they encouraged me to try breastfeeding. At the beginning, they told me to try to breastfeed every two hours. It was smooth initially. My baby was willing to suckle at the beginning, but it only lasted till the first few days… he was not very good at suckling. So, I did go back to the lactation nurses to (let her) teach me again…”* (BF05)

Family support, in particular partner support, facilitated mothers to learn how to breastfeed after returning home from the hospital.

“*My husband was always supportive. At the beginning, my baby seemed to find it difficult to intake breast milk. It's like he gets hungry quickly and soon after he would like to eat again. Then my husband thought that it was too hard for me, and he didn't want us to have irregular sleeping time. So, he studied how to let the baby get the breast milk and searched for methods that I can use to succeed in breastfeeding by the end. Therefore, he is really helpful on this matter.”* (BF19)

However, while mothers appreciated family support, some also indicated a feeling of pressure when the family showed concerns about their difficulties in breastfeeding which was perceived as a discouragement for breastfeeding.

“*At that time, I felt the most confused and I didn't have that experience yet. And then my mother-in-law would say like: ‘what if you stop breastfeeding, it is not necessary for you to breastfeed just because others can do it. You have tried your best and it's time to let go.' But then, in fact, I don't know, I just felt really unpleasant whenever you tell me to stop breastfeeding.”* (BF26)

Participants also indicated seeking information from peer mothers, online or offline, which gave them a sense of “being together for the same goal.” The informational and emotional support from peers gave them the confidence to continue breastfeeding.

“*I have always read articles related to breastfeeding from[sic] the internet. In addition, I joined some groups about breastfeeding online. A lot of mothers there have the same goal and so after talking to those mothers in the group, I feel more relaxed and less stressful[sic].”* (BF27)

Several mothers also mentioned receiving care from “Peiyue,” a woman who was trained to provide at-home care for the first 1–3 months postpartum. Participants appreciated the dietary support for improving breast milk supply from “Peiyue” and their support for dealing with blocked ducts or mastitis.

#### 3.1.2. Personal perseverance

Participants acknowledged that breastfeeding was laborious and physically demanding. They understood that the more they breastfed, the more milk supply there would be and emphasized the importance of perseverance in trying breastfeeding supplemented with frequent breast milk expression, manually or using pumps, during the first 1–2 months to stimulate the breasts to produce more milk. Feeling the increasing breast milk supply was a cue to encourage them to continue.

“*There are no specific reasons for successful breastfeeding but your perseverance and persistence. I think I can succeed because I was diligent, but no other specific reasons. I drank soups and water as usual, but the key to my success was that I used the pump for milk expression or breastfed the baby every two to three hours…”* (BF06)

#### 3.1.3. Adjusting expectations for breastfeeding

The expectation of achieving exclusive breastfeeding while feeling uncertain about their body's capacity gave participants high pressure in the initial period. This was described as an “incompatible expectation” by Hauck (2000). Adjusting expectations for breastfeeding to make the expectations more compactable with their physical and mental state was an important strategy to help them relieve from the pressure. For first-time mothers, this involved setting an effortless mindset to begin, that is, to initiate breastfeeding without expectations for how long to breastfeed or the obligation to succeed.

“*I just try breastfeeding. My mindset is that I will breastfeed if I have (breast milk) and stop it if I have no (breast milk)…I haven't plan[sic] for how long to breastfeed. The best scenario is that I can breastfeed for one year, but I don't know (whether I can succeed). I would wait and see and decide what to do.”* (BF15)

For some, adjusting expectations for breastfeeding was also about setting an achievable goal for how long to breastfeed and making the constant adjustment to achieve a longer breastfeeding duration. Usually, achieving the former short-term goals helped mothers to build up confidence in breastfeeding continuation.

“*Before childbirth, I thought that if I could breastfeed then I would keep going. By the time my child was born, I thought I would only keep breastfeeding till[sic] one month because it was quite difficult. But then when it came to one month, it appeared to be ok and so I kept going…”* (BF02)

Failure in achieving exclusive breastfeeding brought the feeling of guilt and caused self-criticism. Some participants made the comparison with mothers who were less “successful” in breastfeeding which helped them to transition from self-criticism to self-acceptance.

“*At the very beginning, I did have that thought, like initially I would question why I had insufficient milk, because I have a lot of friends around me who just gave birth to their child in a similar period, and I would somewhat make comparisons with them. I felt like I somewhat failed, or felt that I was not good enough. Or why am I so bad in[sic] breastfeeding? There are some self-criticisms. But over time, because there are people who have even less milk than I did…hahaha (laugh)…, I started to think that it is ok to just feed how much I have.”* (BF03)

#### 3.1.4. Supplemented feeding as a transition

“Pumping,” mostly with an electronic pump, was a common strategy to cope with perceiving insufficient breast milk supply and insufficient skills for breastfeeding. Seeing breast milk coming out from breasts through “pumping” and their child drinking their breast milk through a bottle or other tools seemed to enhance mothers' confidence about their body's capacity and gave them reassurance that the child was feeding. However, in some cases (two mothers), over-reliance on “pumping” and predominantly feeding their child with breast milk from bottles at the initial stage eventually evolved into exclusive “pumping.”

“*I always used the pump, and in the first one month, I mainly pumped. But later my friends mentioned that feeding from breasts was the best for both the mother and the baby, and it was also good for the future because you didn't need to wash bottles and the feeling was closer. Then …I told my mom and my ‘Peiyue' to help me try breastfeeding. But actually, I think it would be the best if I tried breastfeeding at the very beginning. It seems that a person like me to try breastfeeding in the middle…I think it is difficult for me and my daughter to learn breastfeeding[sic] together in the second month.”* (BF08)

Infant formula use was usually mentioned as a last resort. Of all, 19 of the 30 participants introduced infant formula in the initial period. The use of infant formula could be introduced by the hospital due to dyad separation or in some cases was advised by the health professional due to concern about the infant's health conditions. However, in most cases, participants had the opportunity to decide whether to use it or not. Using infant formula as supplementation was a difficult decision for participants and was usually made after participants had struggled for some time in trying breastfeeding. In general, working mothers appeared to be more relaxed about using infant formula as they perceived that infant formula can be an option if they had no sufficient milk supply after returning to work. However, despite using infant formula, participants were generally prudent regarding the use of infant formula and kept trying breastfeeding and expression of breast milk to transition to feeding infants exclusively with breast milk.

“*I always breastfeed first, to breastfeed as much as possible. Then (if not sufficient), add a little bit infant formula but I would control the amount, possibly…for instance, just add two ounces, and then try to reduce its amount gradually.”* (BF25)

### 3.2. Themes relating to the coping strategies as breastfeeding is getting attuned

All participants entered a stage of getting attuned which was described by Flacking et al. (2021) as a stage when mothers started to trust their body's capacity to meet infants' demand for breast milk and establish positive mutual interactivity, physically and affectively, with their child during breastfeeding. Participants mentioned that they felt more relaxed about breastfeeding in this stage. However, new challenges emerged as breastfeeding continued. These included the more frequent blocked ducts and mastitis as milk supply increased and the disruptions in lives such as sleep disturbance, dietary restriction, unmet medical needs, and the loss of personal time and space. Moreover, breastfeeding in public was another new challenge as breastfeeding continued. As there was somewhat less social support in this stage, strategies for managing these new challenges indicated overall mothers' active self-exploration. Four themes were identified including seeking support from the online mother groups, deliberating medication, adjusting to accommodate breastfeeding and life normalcy, and managing breastfeeding in public.

#### 3.2.1. Seeking support from online mother groups

Most participants joined at least one online mother group during their pregnancy. This can be partly because we recruited some informants from social media. The online mother groups did not only provide support in the initial stage but also after breastfeeding was getting attuned. The online mother groups were appreciated as a more flexible and easily accessible information source that provided more experiential, personally relevant, and caring support for breastfeeding. Merely recognizing that other mothers were also experiencing similar breastfeeding difficulties could provide psychological reassurance. Several participants shared their experience of how other mothers' experiences obtained from the online groups had been helpful for them to solve the duct blockage without seeking medical help.

“*My first time of mastitis, I remember, it[sic] was in April, because it was Easter and there were no doctors on duty. I remember I had a fever for two consecutive days and so I asked the other mothers…We have a WhatsApp breastfeeding group. Many mothers there share their experience[sic]. They taught me to let the baby suckle more if the baby was willing to suckle, to remove the blockage of the ducts. If this doesn't work after one or two days, I would have to see a doctor.”* (BF24)

However, in member checking, one mother raised one concern that seeing other mothers' achievement in breastfeeding in the online group also gave pressure for some mothers in breastfeeding.

#### 3.2.2. Deliberating medication

While participants were more passive in receiving professional support in the initial stage of breastfeeding, they were more active in seeking and choosing the “right” medication that would not affect their breastfeeding when experiencing blocked ducts, mastitis, or other medical problems in this period. As breastfeeding and the baby were viewed as the top priority, some indicated distrust in doctors who somewhat “discouraged” breastfeeding. They were more deliberative when choosing a doctor or deciding whether to take a medication. Due to fear of any drop in breast milk supply caused by the medication, participants tended to avoid Western medicine and favored using traditional Chinese medicine that was perceived to be milder or self-medication (e.g., dietary therapy, massage, or more frequent breastfeeding or pumping) to solve their medical needs or even delayed unurgent medical needs.

“*And then I had rubella (German Measles), to be honest at that moment milk supply was easy to decline. But because I am still lactating, I didn't go to a Western doctor. I was afraid that he/she would prescribe steroids. I went to see a Traditional Chinese Medicine practitioner instead. The practitioner taught me regimens of diets and prescribed the[sic] medication that was very mild. Just some medicine for me to wash my body. And then for the whole month, I only had steamed pork ribs, steamed fish, and other steamed foods. It doesn't affect milk production and the baby seems to be happy and so I was able to keep on breastfeeding.”* (BF13)

#### 3.2.3. Adjustment to accommodate breastfeeding and life normalcy

Participants perceived that the time and commitment input for breastfeeding caused disruptions to various aspects of life such as sleep problems and dietary restrictions. For working mothers, sleep disruption can affect their work the next day, which is particularly challenging. They felt that they were “tied to” or “trapped in” breastfeeding, physically and mentally, which means loss of personal time and space for personal hobbies, social interactions, and intimate activities with their partner. Adapting to breastfeeding continuation means that they need to actively adjust their lives to accommodate breastfeeding. Overall, most participants would compromise their personal needs in life for the sake of breastfeeding and felt that this was worthwhile as they perceived the benefits of breastfeeding to their child. While there was usually a difficult period when adjusting to their lives, most indicated physically and mentally capable of finally adapting to the changes.

“*Yes, it is very tiring, but it is OK after you get used to it. Once in a while, the baby would wake up many times at night, then I would be very tired the next day. But usually, he wakes up 2-3 times a night. I think that is acceptable. As long as you can get used to it, it's OK.”* (BF25)

Participants emphasized the importance of active arrangements to integrate breastfeeding into life routines.

“*You arrange your own time. For example, after work, if you have to go out, you need to pump milk or breastfeed before you go out, I mean to put breastfeeding into details of your life routines…yes…that is, making it a part of your habits.”* (BF28)

The adjustment was sometimes also made for breastfeeding to accommodate personal needs, particularly among participants who were nursing their second child.

“*I can get used to the changes in diets. But sometimes I would drink coffee. People were concerned about the caffeine or things like that (in the coffee). But now I sometimes would have one cup a day. I try my best not to drink it, but for my second child, I am a bit more reckless because sometimes I feel really tired, and really can't endure the tiredness and want to drink coffee.”* (BF29)

#### 3.2.4. Managing breastfeeding in public

Participants generally perceived insufficient baby-care facilities in public places. Although most participants perceived increasing social acceptance of breastfeeding in public, some mentioned occasional unpleasant experiences due to others' negative responses or comments. Three tactics to manage progressively evolved and helped participants adapt to breastfeeding in public.

Avoidance: This represents a more passive coping approach. Participants who were first-time mothers tended to avoid going out or carefully plan for the time of going out to avoid breastfeeding in public.

“*You need to line up to wait for the nursery rooms in shopping malls. You need to lie down to breastfeed. You know it's different to breastfeed at home and outside. The baby is not very cooperative (when breastfeeding outside) and there are more troubles. I seldom go out.”* (BF10)

Dismissing: While participants avoided breastfeeding in public, such a strategy evolved into dismissing or distancing, meaning that they tried to care less about others' views as they perceived an urgent need to feed their child in public.

“*At this moment (when the child is crying) you have to breastfeed, you have to tolerate and not to care others' views too much. When there is no one watching you…actually nothing to be watched… but if they want to watch you, you can do nothing about it. So, I became really thick-skinned for this.”* (BF15)

Using nursing scarves: This was a more proactive coping strategy. Most participants eventually accepted breastfeeding in public with a nursing scarf following dismissing others' unfavorable views.

“*I would breastfeed as usual even in public areas, but of course with a scarf on. I would just go ahead to breastfeed without worrying about anything.”* (BF12)

### 3.3. Themes relating to coping strategies for returning to work

Working mothers raised major concerns about returning to work including employers' negative views, co-workers' complaints, busy working schedules, insufficient facilities to support breast milk expression in the workplace, the decline in milk supply, and difficulty for the child to get used to bottle feeding after returning to work. Strategies for managing concerns and challenges for breastfeeding continuation after returning to work were centered around active preparation and arrangement for breast milk expression in the workplace. These included planning ahead, gaining workplace support, and proactive adjustment for breast milk expression.

#### 3.3.1. Planning ahead

Preparation for continued breastfeeding after returning to work had been made 2 to 3 weeks before participants returned to work. These included preparing pumps and other tools for breast milk expression in the workplace [e.g., “*I brought the milk pump, bottles, and the ice bag (before returning to work)”* (BF24)]; expressing and storing extra breast milk [e.g., “*Before returning to work, I had already pumped a lot of milk”* (BF18)]; and gaining family support for feeding the baby [e.g., “*I have to teach them (my family) how to freeze the milk, remind them of checking the expiry date, yes, and teach them how to warm the milk as well as the right temperature”* (BF28)]. Good preparation appeared to relieve mothers' stress about returning to work.

#### 3.3.2. Gaining workplace support

While workplace support was essentially important for mothers to continue breastfeeding after returning to work, for participants, it was not always “given” but sometimes required negotiation with the employers.

“*I had talked to my company about this (I need to pump milk in the company) before returning to work. Fortunately, the company can coordinate this.”* (BF20)

Even when employers' approval had been obtained and the facilities were available for breast milk expression, participants mentioned that they still needed to put in personal efforts or make adjustments to use those facilities and spare time for breast milk expression.

“*I had talked to my manager before I go[sic] back to work that I would continue breastfeeding, and that I may need a bit longer lunch break for pumping milk. And he was very nice and quickly approved, but they did not tell me where I can pump milk. I need to find the place myself.”* (BF23)

#### 3.3.3. Proactive adjustment for breast milk expression in the workplace

Despite workplace support, all working mothers indicated the importance of proactive adjustment to balance the needs of work and breast milk expression. The adjustment included a series of proactive strategies ranging from self-management of time for milk expression breaks, to adjustment of their physical body to adapt to the working schedule, and re-organizing working hours for completing work-related tasks.

“*You need to adjust the time. For example, I have fixed the time to pump milk to be at 10 o'clock every day, but I can't make it because I have a meeting at 10 o'clock. Then, I should expect that the meeting will always be late but not early, and I would go to do pumping at 9 o'clock myself to finish it before the meeting.”* (BF08)

“*What matter is the arrangement of my personal work. Because it is possible that I have used one hour for milk expression, but you have to consider your work. Or…you should stay a bit late to finish your work before going home…that is, you personally need to arrange your work and complete it.”* (BF20)

### 3.4. Themes relating to cues to breastfeeding continuation

While a lot of cognitive efforts were needed to help participants overcome challenges experienced during their breastfeeding journey, cues that help to maintain breastfeeding were overall affective, experiential, internal and norms-related, and automatically prompt breastfeeding continuation. Participants overall indicated that all their efforts to breastfeeding were worthwhile, or even perceived that the process was “sweet” or “amazing” rather than painful after they had overcome the initial difficulties. This indicates that it is overall those positive experiences and emotions that facilitate breastfeeding to continue. Seven themes relating to cues to breastfeeding maintenance emerged.

#### 3.4.1. Enjoyment, intimacy, and closeness

All participants who eventually achieved breastfeeding indicated that they enjoyed breastfeeding. The breastfeeding moment was described as “sweet,” “affectional,” “happiest,” and “extraordinary.” Such moment was contributed by the physical contact with their child (e.g., holding the infant) and the positive responses of the infant (e.g., being calm, like suckling), which created positive emotional experiences.

“*My initial intention may change, but it always come[sic] to the same decision, that is, it is not good to stop, I should continue to breastfeed. I would breastfeed as far as I have milk because you are unwilling to give up the affectional feeling that the child is clinging to your body. It makes you very happy.”* (BF12)

For working mothers, breastfeeding provided an opportunity to build up a connection with their infant, physically and emotionally. Such embodied emotions appear to make breastfeeding continuation a default choice for mothers because for them, stopping breastfeeding was perceived as a kind of “loss” and “take away” their “happiness.”

“*After I leave my work and come back home, I would breastfeed. When I hold my baby in my arms to breastfeed her, this means something I think, like after I finished work, I can see my baby and I can hold her, she is safe and having milk. For me, this is the very happy thing…my happiest moment in a day.”* (BF04)

#### 3.4.2. Breastfeeding as a personal achievement

Being “successful” in breastfeeding seemed to enhance mothers' sense of personal values and their confidence in the accomplishment of a task like breastfeeding, and a source of “pride.” This is particularly important for working mothers who had fewer opportunities to enjoy breastfeeding.

“*I feel that I'm so amazing, yes, it is really amazing. I have never thought…Before I was married, I have never thought about[sic] that I would be so determined for completing a task like breastfeeding. I'm not that kind of persons[sic] who are[sic] very perseverant. However, for breastfeeding, I can make it naturally and I was successful. And I have the motivation to keep going. It almost went out of my expectation.”* (BF28)

This indicates that “success” in breastfeeding is viewed as a kind of personal achievement which encourages breastfeeding continuation. As illustrated in the below quote, mothers' conceptualization of breastfeeding success was somewhat different from the medical standards, which had an emphasis on their satisfaction with the outcome (e.g., to satisfy their child's need) rather than meeting those objective medical metrics (e.g., mode, duration, and exclusivity).

“*I think it is you found that…for instance, you pump milk, and you can pump a lot. Then other people see this, they would say, oh, you can pump so much. At that moment, indeed I would feel a bit proud. That is, even if I just pump, I make it! Yes.”* (BF14)

#### 3.4.3. Healthy and thriving child

Participants perceived that a healthy and thriving child was the outcome of their efforts input for breastfeeding and hence a sense of accomplishment and a source of “joyfulness.” A healthy and thriving was a particularly important cue for working mothers to maintain active “pumping” after returning to work.

“*It is worthwhile even if it is difficult because, without such sacrifice, I would not be able to keep the amount (of milk production) and the healthiness of my son. I feel that the outcome is satisfying, being healthy, [sic]good amount of my breast milk, and that he is breastfed well and get[sic] full.”* (BF14)

Most participants perceived that their child had “better immunity” and believed that this was due to their efforts to transfer the “goodness” to their children through breastfeeding. The “better immunity,” according to participants, was indicated by “no fever” following childhood vaccination, “quick recovery” after getting an infection, and overall “fewer sicknesses.”

“*His immunity is really good. For example, whenever he took vaccination, some children would have a fever, in fact, he would also have a fever, but it would be a low fever so that there was no need for an antipyretic drug.”* (BF19)

Such positive experiences drove participants to self-confirm their breastfeeding efforts and motivate them to continue breastfeeding for a longer period to help children overcome key development milestones.

“*I really wanted to give up at 13 months, but I really wanted to make it till he is one and a half years when he needs to take another vaccination.”* (BF13)

#### 3.4.4. Positive social cues

Positive social cues were those positive feedback from significant others (e.g., health professionals and family) and general others about mothers' breastfeeding performance. Those positive social feedback helped to clarify participants' uncertainty about their breastfeeding performance and gave strong emotional support for their persistence in breastfeeding.

“*I had once questioned myself for a long time. But then I went to the Maternal and Child Health Centres, the nurse at the centre said you had already been pretty good at breastfeeding. You can breastfeed for such a long time.”* (BF027)

“*People would say: you are so good [sic]a mother that you make him strong and white! That is, he has been fed so well. Also, people would appreciate your perseverance, and you would feel people's recognition and appreciation. I do not mean that I want their praise, but I would get their support and appreciation because of my perseverance.”* (BF13)

The positive social feedback also shaped the social norms of accepting long-term breastfeeding. Participants seemed to feel more uncertain about whether they should continue breastfeeding for a longer period (e.g., beyond 6 months). Knowing other mothers' breastfeeding continuation for a long period helps clarify the norms and enhance their confidence to achieve the goal of longer breastfeeding duration.

“*Initially, I wanted to breastfeed until six months. But then I saw on a Facebook breastfeeding group that some mothers would breastfeed till the age of two or three years… After reading this, I have the feeling that oh they can breastfeed for such a long time and mothers can be so great! And then my mindset changed, and I did not put a limit about[sic] when to stop breastfeeding. Whenever the baby wants to stop then she can stop.”* (BF23)

#### 3.4.5. Bodily response

Participants mentioned constantly monitoring their bodily responses during their journey of breastfeeding to decide whether to continue breastfeeding. In the initial stage, actively trying breastfeeding and “pumping” was encouraged to be continued due to feeling an increase in breast milk supply. As breastfeeding continued, feeling that they still had breast milk supply usually reminded them of their capacity as a mother to give “goodness” to their child and thereby an important cue to decide whether to stop or continue breastfeeding.

“*I had not thought about giving up…Because…Uh… I would continue to monitor whether I still have milk or not. Yes. If I completely have no milk, I mean if I cannot pump out any milk, then I would give up. But in fact, I can keep pumping out some. So, I just kept going, because I just want the baby to have breast milk as much as possible.”* (BF03)

#### 3.4.6. Enjoying the convenience of breastfeeding

Participants who eventually achieved breastfeeding appreciated the convenience of breastfeeding compared with infant formula. For them, breastfeeding cessation means that they would have to switch to formula feeding which was perceived as an inconvenient option having to prepare infant formula and hence enhanced the positive emotion about breastfeeding continuation. For these mothers, breastfeeding continuation was a preferred choice while discontinuation means “losing” the convenience of breastfeeding. The convenience of breastfeeding was a particularly important cue for unemployed mothers to maintain breastfeeding.

“*Why did I want to keep breastfeeding so much? It is because I think that handling milk powder is too annoying. You have to bring a lot of things with you when going out. You need to prepare a lot of things and then when the baby needs to have milk, for instance, if the baby cries, you have to go through the process of preparing the bottle of infant formula. Actually, breastfeeding is very convenient. You just immediately breastfeed the baby and you can quickly calm her. This is a reason why I persist breastfeeding.”* (BF11)

#### 3.4.7. Breastfeeding as a motherhood commitment

Several participants described that the period for breastfeeding was “special” and temporary for both the mother and their children. Despite perceiving some personal sacrifices, they perceived that it was worthwhile because efforts input for 1–2 years could help the child to gain long-term benefits. These thoughts indicate a process of weighing up the costs and benefits of breastfeeding but also internally link to the norms that it is a motherhood commitment to preserving breastfeeding as long as possible.

“*Think about it, how long can the baby have breast milk? At most one or two years. You just need to work hard for one or two years for the baby and it is worthwhile. This is what should be done as a mother.”* (BF10)

## 4. Discussion

This qualitative study revealed themes relating to strategies and cues that helped mothers to maintain breastfeeding. All participants had proactively engaged in developing their strategic plans for coping with the challenges throughout their breastfeeding journey. Their coping strategies were overall dynamic, involving a process of constant adjustment, proactive self-exploration, and planning. In the initial stage, participants relied more on various social support to initiate breastfeeding. The baby-friendly hospital policy was particularly emphasized as an essential support for mothers in knowledge preparation, obtaining breastfeeding skills and addressing concerns about children's health conditions and when mothers felt unsure about whether breastfeeding should be continued. Breastfeeding initiation was generally perceived to be difficult, and personal perseverance in “trying” breastfeeding and active “pumping” to stimulate breast milk supply was emphasized by participants. Feeling the bodily response of increased milk supply was an encouragement for their perseverance. During breastfeeding initiation, unrealistic expectations for breastfeeding induced pressure on mothers, which may undermine breastfeeding success (Whipps et al., 2021). Adjusting expectations for breastfeeding was commonly emphasized by the participants to help them gradually achieve longer breastfeeding. Other studies also reported that mothers used strategies of “lowering demand,” “keeping an effortless mindset,” and “having realistic expectations” to address the negative emotions (e.g., “guilty” and “personal failure”) associated with the pressure of exclusive breastfeeding (Lyons et al., 2019; Jacobzon et al., 2022). “Pumping” was commonly mentioned as a coping strategy for insufficient skills in breastfeeding, perceiving insufficient milk supply, and feeling unconfident about their body's capacity in breastfeeding. In Hong Kong, over 80% of mothers reported feeding their infants with expressed human milk within 6 months postpartum (Pang et al., 2017). Seeing that breast milk was pumped out from the breasts and that their child was drinking the expressed breast milk via a bottle gave them confidence about their body's capacity. Infant formula was usually a last resort and used with prudence. Participants who supplemented “pumping” and infant formula use with predominant breastfeeding generally can transition to exclusively feeding their child with breast milk. However, those who over-relied on supplementary feeding may either become exclusive “pumping” or prolonged the period of transition to exclusive breastfeeding.

Even though breastfeeding was perceived to be laborious and effortful, all participants eventually entered a stage of getting attuned when they were more confident about their body's capacity in breastfeeding. As there was somewhat less social support in this stage, mothers were more proactive in seeking the right support, deliberating medication due to concern that breast milk supply may decline, adjusting to balance the needs for breastfeeding and lives and managing breastfeeding in the public. These strategies indicate that while breastfeeding remains the top priority for mothers in this stage, some adjustments are made to accommodate their personal needs and make breastfeeding more compatible with their lives, for instance, being more relaxed about their diets and breastfeeding in public (e.g., with a nursing scarf). These adjustments reciprocally benefit breastfeeding continuation. As breastfeeding was getting attuned, mothers became more positive about breastfeeding. The positive-experienced emotions, such as feeling “sweetness,” “enjoyment,” and “pride,” about breastfeeding were important cues that help to maintain breastfeeding. However, these positive emotional experiences and their roles in cueing breastfeeding continuation were generally overlooked in existing literature (Zhao et al., 2018; Tang et al., 2019; Patil et al., 2020). The convenience of breastfeeding, once it was achieved, was also emphasized and emerged as a cue to make breastfeeding a preferred choice. However, these cues were irrelevant for mothers who predominantly fed their child with expressed breast milk and the working mothers who had to maintain breastfeeding through “active pumping” in the workplace. For working mothers and mothers who predominantly “pumped,” “personal achievement,” “a healthy and thriving child,” and “positive social feedbacks” were the more relevant cues. This indicates that cues to encourage breastfeeding continuation should be tailored by how breast milk is given to infants, mothers' values, and the stage of breastfeeding.

For working mothers, our study found that being active in making preparation and arrangement for breast milk expression in the workplace was essential to maintain breastfeeding after returning to work. Existing literature has documented the importance of workplace and employers' support for promoting breastfeeding duration (Dinour and Szaro, 2017; Wallenborn et al., 2019). Our study further suggests that even when the workplace and employers' supports are available, participants still need to make an effort to negotiate with their employers, actively make adjustments for using the facilities and lactation breaks at the workplace, and reorganize their working hours to meet the need for work.

Overall, our study findings were comparable to existing studies that focused on mothers' positive experiences and positive emotions with breastfeeding (Lyons et al., 2019; Flacking et al., 2021; Jacobzon et al., 2022). However, compared with existing studies, our study further illuminates the contexts such as how strategies for creating positive experiences with breastfeeding would be varied by stage of breastfeeding and how cues to breastfeeding continuations can be differed by mothers' employment status. Existing studies of similar topics mainly focused on mothers or babies with special health conditions (Lyons et al., 2019; Flacking et al., 2021). Our studies suggested that some of the coping strategies such as “realistic expectations for breastfeeding” used by participants with special health conditions can also be applied to healthy mothers and babies.

## 5. Study limitations

Our study was somewhat interrupted by the COVID-19 pandemic. Despite this, all participants for whom the interviews were conducted during the pandemic indicated the little impact of the pandemic on their breastfeeding except for more opportunities to breastfeed at home and being more careful about the sanitation of the settings before breastfeeding or “pumping.” The latter 12 interviews were conducted via telephone rather than face-to-face due to the pandemic, which hindered collecting the non-verbal information (e.g., participants' facial expressions) during those interviews. In addition, although most interviews were conducted in a relatively quiet environment (e.g., an office room or park), three interviews were conducted in a relatively noisy environment (e.g., canteen or fast food shop) for participants' convenience. However, these three participants' willingness to share was not affected by the setting of the interview. Furthermore, most of our participants had breastfed for more than 6 months. Therefore, there may be recall bias when they were asked to recall how their breastfeeding was initiated though most participants mentioned that the memory remained vivid. There may also be biases arising from how informants were recruited and the characteristics of our sample. First, since this study focused on the strategies for and cues to breastfeeding maintenance among mothers who were “successful” in breastfeeding maintenance, it provided limited insights into why others could not achieve breastfeeding maintenance. Second, informants who were recruited using social media may tend to emphasize the importance of online mother groups in supporting breastfeeding. Third, most of our participants had a tertiary or above educational attainment. However, it was estimated that over 60% of the women aged 18–44 years in Hong Kong had an educational attainment of tertiary or above (Hong Kong Census Statistics Department, 2023).

## 6. Trustworthiness of the study

Several strategies had been employed to ensure the trustworthiness of the research findings. First, the phenomenological framework was chosen to guide the study procedure. Second, peer debriefing was conducted by involving researchers who had no previous research interest in breastfeeding to check the transcripts and the codes. All codes, categories, and themes were iteratively discussed among the research team until a consensus was reached. Third, member checking was employed to invite three participants to give feedback on the reports. All invited participants provided positive feedback on the reports and agreed that the themes were relevant to their breastfeeding experiences.

## 7. Conclusion

Overall, our study indicates that breastfeeding maintenance is both a laborious and effortful process during which psychosocial support and mothers' dedication, adjustment, and self-exploration are involved, and an affective and cue-based process during which cues emerge from their positive experiences and positive feelings about breastfeeding. Interventions to encourage breastfeeding maintenance should aim for facilitating these dual processes. On the one hand, mothers should be mentally prepared for the difficulties of breastfeeding particularly in the initial stage to avoid unrealistic expectations about breastfeeding. However, they should also be encouraged that breastfeeding will always get easier as they persevere. On the other hand, breastfeeding is embodied with various positive cues arising from their breastfeeding experiences. These cues play an important role in empowering mothers for breastfeeding maintenance. Cueing interventions have been suggested to be effective in promoting daily health routines such as diets and hand hygiene (King et al., 2016; Papies, 2017). Maintenance of breastfeeding requires mothers to make breastfeeding as a part of their life routines. Future studies should consider integrating relevant cues into existing psychosocial interventions for promoting breastfeeding maintenance.

## Data availability statement

The raw data supporting the conclusions of this article will be made available by the authors, without undue reservation.

## Ethics statement

The studies involving human participants were reviewed and approved by the Institutional Review Board of the University of Hong Kong (Reference No.: UW19-293). The patients/participants provided their written informed consent to participate in this study.

## Author contributions

QL conceived and designed the study, conducted the interviews, analyzed the data, and drafted the manuscript. JY assisted in subject recruitment and data collection and analyzed the data. KL and SN coordinated and facilitated subject recruitment and provided expertise in the study design. YC assisted in data collection and checked and translated the quotes for illustration. WW provided expertise in study design and data analysis. All authors have reviewed the draft and provided critical input. All authors approved the final manuscript.
