# Supplementary material for: Learning from mothers' success in breastfeeding maintenance: coping strategies and cues to action
Source: Front Psychol. 2023 May 16;14:1167272. doi: 10.3389/fpsyg.2023.1167272 (PMC10229069; doi:10.3389/fpsyg.2023.1167272)
Supplement: Supplementary file 2 [file Table_1.DOCX]

Supplementary Material

Learning from mothers’ ‘success’ in breastfeeding maintenance: coping strategies and cues to action

Qiuyan Liao*, Jiehu Yuan, Kris Yuet Wan Lok, Siew Fei Ngu, Yuyi Chen, Wendy Wing Tak Lam

*** Correspondence:** Qiuyan Liao: qyliao11@hku.hk

# Supplementary Table: Characteristics of the participants

| ID | Age (years) | Educational attainment | Family income (HK$)^a^ | Working status | Number of children | Child’s age (months)^b^ | Patterns of breastfeeding |
| --- | --- | --- | --- | --- | --- | --- | --- |
| BF01 | 33 | Tertiary | Refused to answer | Part-time | 1 | 9 | Introduced infant formula for the first 1-2 days; then exclusively breastfed with occasional milk expression; still breastfed |
| BF02 | 31 | Tertiary | 40,000-60,000 | Full-time | 1 | 8 | Exclusively breastfed before returning to work and combined breastfeeding and milk expression after returning to work; still breastfed |
| BF03 | 25-34 | Master or above | ≥60,000 | Full-time | 1 | 11 | Breastfed with formula supplementation until 6 months; then transitioned to exclusive breastfeeding with complementary solid food; still breastfed |
| BF04 | 25-34 | Tertiary | 30,000-40,000 | Full-time | 1 | 6 | Breastfed with formula supplementation until 5 months; then transitioned to predominantly breastfeeding and occasional milk expression; still breastfed |
| BF05 | 35-44 | Tertiary | 40,000-60,000 | Ful-time | 1 | 8 | Breastfed with formula supplementation until 1.5 months; frequent expressed breast milk in the first two months to stimulate milk production; transitioned to a combination of breastfeeding and milk expression after 1.5 months; still breastfed |
| BF06 | 32 | Tertiary | 40,000-60,000 | Full-time | 2 | 8 | Predominantly breastfed and occasionally expressed milk; more frequent milk expression after returning to work; still breastfed |
| BF07 | 32 | Tertiary | ≥60,000 | Full-time | 3 | 4 (twins) | Exclusively breastfed for her twins before returning to work; combined breastfeeding and milk expression after returning to work; still breastfed |
| BF08 | 35 | Master or above | ≥60,000 | Full-time | 1 | 8 | Introduced infant formula for the first several days; then combined breastfeeding and milk expression until 2-3 months; thereafter, transitioned to exclusive milk expression due to child’s refusal of breastfeeding and returning to work; still expressed milk |
| BF09 | 30 | Secondary | 20,000-30,000 | Unemployed | 2 | 10 | Exclusively breastfed throughout; still breastfed |
| BF10 | 33 | Secondary | 20,000-30,000 | Unemployed | 1 | 8 | Exclusively breastfed throughout; still breastfed |
| BF11 | 31 | Tertiary | 20,000-30,000 | Unemployed | 2 | 10 | Breastfed the 1^st^ child during pregnancy and continued after delivering the 2^nd^ child; exclusive breastfeeding; still breastfed the two children |
| BF12 | 40 | Secondary | 30,000-40,000 | Unemployed | 4 | 18 | Exclusively breastfed throughout; still breastfed |
| BF13 | 35-44 | Tertiary | ≥60,000 | Full-time | 2 | 24 | Introduced infant formula initially; then combined breastfeeding and milk expression and continued this mode after returning to work; breastfed until the child was 18 months old |
| BF14 | 25-34 | Tertiary | 40,000 | Full-time | 1 | 13 | Combined breastfeeding, milk expression and formula supplementation in the first month; transitioned to a combination of breastfeeding and milk expression thereafter; became exclusive milk expression since the fourth month due to child’s refusal of breastfeeding; still expressed milk |
| BF15 | 34 | Tertiary | 30,000-40,000 | Unemployed | 2 | 7 | Breastfed with formula supplementation for the first two weeks and gradually transitioned to a combination of predominant breastfeeding and occasional milk expression; still breastfed |
| BF16 | 35 | Tertiary | 30,000-40,000 | Unemployed | 2 | 6 | Breastfed with formula supplementation for the first one month; expressed milk to stimulate milk supply; transitioned to a combination of breastfeeding and milk expression after the first month; still breastfed |
| BF17 | 38 | Master or above | ≥60,000 | Part-time | 2 | 7 | Breastfed with formula supplementation in the first one month; transitioned to a combination of predominantly BF and occasional milk expression thereafter; still breastfed |
| BF18 | 25-34 | Secondary | 10,000-20,000 | Full-time | 1 | 18 | Breastfed with formula supplementation for the first several days; transitioned to a combination of breastfeeding and milk expression; still breastfed |
| BF19 | 35-44 | Tertiary | ≥60,000 | Full-time | 1 | 22 | Breastfed with formula supplementation for the first two months; transitioned to exclusive breastfeeding thereafter; combined breastfeeding and milk expression after returning to work; still breastfed |
| BF20 | 25-34 | Master or above | ≥60,000 | Full-time | 1 | 18 | Breastfed with formula supplementation for the first month; exclusively breastfed since the second month; kept expressing milk to stimulate milk supply; combined breastfeeding and milk expression after returning to work; still breastfed |
| BF21 | 25-34 | Tertiary | 10,000-20,000 | Full-time | 2 | 6 | Breastfed the 1^st^ child during pregnancy, and continued after delivering the 2^nd^ child; breastfed the 1^st^ child for 26 months and the 2^nd^ child for 6 months; exclusive breastfeeding; still breastfed both children |
| BF22 | 35-44 | Tertiary | ≥60,000 | Unemployed | 1 | 20 | Mostly breastfed and occasionally expressed milk; breastfed the child for 19 months; had stopped breastfeeding for one month |
| BF23 | 25-34 | Tertiary | 20,000-30,000 | Part-time | 1 | 11 | Breastfed with formula supplementation at the beginning; then transitioned to a combination of exclusive breastfeeding and occasional milk expression; still breastfed |
| BF24 | 34 | Secondary | ≥60,000 | Full-time | 1 | 14 | Introduced infant formula in the hospital; Predominantly expressed milk and occasionally tried breastfeeding for the first three weeks; then transitioned to mostly breastfeeding and occasional milk expression; still breastfed |
| BF25 | 35-44 | Master or above | ≥60,000 | Full-time | 3 | 11 | Breastfed with formula supplementation initially; then transitioned to a combination of mostly breastfeeding and occasional milk expression after returning to work; still breastfed |
| BF26 | 35-44 | Tertiary | ≥60,000 | Full-time | 2 | 19 | Breastfed with formula supplementation for the first several months due to insufficient milk supply; manually expressed breast milk to stimulate milk production; stopped formula after introducing solid food; still breastfed |
| BF27 | 32 | Master or above | 30,000-40,000 | Full-time | 1 | 7 | Combined breastfeeding, milk expression and formula feeding for the first one month; then transitioned to exclusive breastfeeding but combined breastfeeding and milk expression again after returning to work; still breastfed |
| BF28 | 25-34 | Tertiary | ≥60,000 | Full-time | 2 | 10 | Predominantly breastfed and occasionally expressed milk; still breastfed |
| BF29 | 25-34 | Master or above | 40,000-60,000 | Unemployed | 2 | 15 | Exclusively breastfed throughout; still breastfed |
| BF30 | 35-44 | Secondary | 30,000-40,000 | Unemployed | 2 | 6 | Breastfed with formula supplementation at the beginning and then transitioned to exclusive breastfeeding; had breastfed the child for six months; just stopped breastfeeding |

^a^ 1HK$ = 0.13US$

^b^ The youngest child of the participants

**Interview guide**

*Objectives*:

1. To explore the challenges encountered by Hong Kong mothers during breastfeeding and their coping strategies
2. To explore cues that help to sustain breastfeeding

*Self-introduction*:

Hello, Ms. …, I’m … from…. Thank you for being willing to participate in this interview. The interview format will be just like talking to a friend about your experience and beliefs regarding breastfeeding. During the conversation, I will ask you several questions to understand your difficulties, how you overcome those difficulties during breastfeeding and your beliefs about breastfeeding. There are no right or wrong answers for all my questions. You just need to share with me on these aspects based on your actual experience, thoughts and feelings. The information you provided will be very helpful for us to guide public health interventions for promoting breastfeeding. The whole process will be audio-taped. All data will be kept anonymous. Do you have any questions about this study before we start the interview?

Then I will open the recorder to start the interview….

*Questions used to guide the in-depth interview*:

1. How many children do you have? How old are he/she/they? What was the delivery method? How about breastfeeding for your children/child? (How long have you breastfed your child/each child? Is it exclusive breastfeeding?)
2. For this child/youngest child, did you plan for breastfeeding before delivery? Why? Are there any other reasons? Did you have any plan for how long you would breast feed this child? How was your plan? Why did you have such plan?
3. What difficulties did you encounter when you initiate breastfeeding? Could you please tell me more details about that? How did you cope with this difficulty? Enventually, what made you to insist on breastfeeding?
4. After you have overcome those difficulties when you initiate breastfeeding, did you encounter other difficulties? What were they? How did you cope with those difficulties? Did you have a thought of giving up breastfeeding when you encountered those difficulties? Why or why not? What were the reasons for you to insist on breastfeeding even though it was not easy for you?
5. (For working mothers) When did you return to work postpartum? Did you plan to breastfeed after returning to work? Why or why not? What were your concerns about breastfeeding after returning to work? What difficulties did you experience after returning to work? How did you cope with those difficulties? What were the major reasons for you to insist on breastfeeding after returning to work even though there were difficulties?
6. During your journey of breastfeeding, what do you enjoy most? Could you please give more details about your thoughts and feelings?
7. What benefits or the positive things you experience during breastfeeding? Could you please tell me more about those experiences?
8. Overall, what are the major reasons for you to insist on breastfeeding? Among all these reasons, which are more important for you? Why?
9. Overall, what do you want to achieve through breastfeeding? Why? How do you feel about the whole process of breastfeeding?
10. Do you have any other things to share with me regarding breastfeeding?
